# Circadian Rhythm and Lipid Metabolism: The Role of the Gut Microbiota

**DOI:** 10.3390/life16101725

**Published:** 2026-10-15

**Authors:** Xinyu Lv, Huixin Liu, Mengkuan Liu, Chunxin Liu, Yixing Li, Jie Ma

**Affiliations:** Guangxi Key Laboratory of Animal Breeding, Disease Control and Prevention, College of Animal Science and Technology, Guangxi University, Nanning 530004, China; lvxinyu20242024@163.com (X.L.); 2518390125@st.gxu.edu.cn (H.L.); a19103878939@163.com (M.L.); 13633069052@163.com (C.L.); liyixing39@gxu.edu.cn (Y.L.)

**Keywords:** circadian rhythm, lipid metabolism, gut microbiota, hormones

## Abstract

Circadian rhythms play a vital role in lipid metabolism by coordinating lipogenesis, lipolysis and fatty acid oxidation to maintain energy homeostasis. Their regulation is affected by light exposure, feeding time, dietary composition, hormonal signals and gut microbiota. Accordingly, persistent circadian misalignment may therefore increase susceptibility to obesity and related metabolic disorders. Increasing evidence also indicates that the gut microbiota exhibits marked diurnal variation and can, in turn, modulate host metabolic rhythms through microbial metabolites and host–microbe signaling. This review summarizes the major factors influencing circadian rhythms, discusses their regulatory effects on lipid metabolism and the contribution of the gut microbiota, and outlines the relationship between circadian disruption and obesity, with the aim of providing insights into the prevention and intervention of metabolic diseases.

## 1. Introduction

Circadian rhythms are widely conserved across living organisms and are crucial for maintaining internal homeostasis and adapting to cyclical changes in the external environment [1]. They rely on an endogenous circadian clock system to organize temporal information within the body and modulate a broad range of physiological processes, including sleep rhythms, metabolic activity, emotional fluctuations, hormone synthesis and secretion, and immune function, thereby supporting the orderly coordination of biological activities [2]. As artificial light at night, shift work, sleep deprivation, and irregular eating patterns have become increasingly common, normal temporal organization has become more vulnerable to disruption. Epidemiological studies have linked shift work and social jetlag to weight gain and an increased risk of cardiometabolic disorders [3,4]. Lipid metabolism is strongly influenced by time of day. Fatty acid synthesis and oxidation, triglyceride storage and mobilization, and cholesterol and bile acid metabolism are jointly regulated by the circadian clock, nutritional status, and endocrine signals [5]. When the temporal coordination of these processes is disrupted, lipid uptake, utilization, and storage may become imbalanced, which can subsequently affect the function of the liver, adipose tissue, and other metabolic organs. Therefore, abnormalities in lipid metabolism cannot be fully understood by considering energy intake or individual metabolic pathways alone; their temporal context must also be taken into account.

In recent years, the gut microbiota has added a new dimension to this field. Its composition and metabolic activity are not static but fluctuate over the course of the day in response to light exposure, feeding, and changes in host physiology [6]. Microbial metabolites, including short-chain fatty acids, bile acids, and tryptophan derivatives, can also interact with host metabolic and circadian signaling pathways [6,7]. The relationship between circadian rhythms and lipid metabolism is shaped not only by host regulatory mechanisms, but also by the gut microbiota and its metabolic activity. Circadian rhythms, the gut microbiota, and lipid metabolism have each been studied extensively, but the connections among these systems remain less clearly integrated, particularly the role of the gut microbiota in linking host temporal regulation with lipid homeostasis. This review focuses on how changes in host rhythmicity influence microbial composition and metabolic activity, and how microbial signals may in turn affect peripheral clocks and lipid metabolism. From this perspective, we further examine how these interactions relate to obesity and associated metabolic disorders, while considering the directionality and causal relationships that remain unresolved.

## 2. Circadian Rhythm

The Earth’s rotation generates regular light–dark cycles, and most organisms have developed circadian rhythms that are synchronized with the external environment through long-term adaptations [8]. These rhythms are regulated by endogenous biological clocks, causing physiological and behavioral activities to exhibit approximately 24-h cycles [9]. Circadian regulation coordinates internal physiological processes with predictable environmental changes and contributes to the maintenance of homeostasis [10]. This temporal organization provides the basis for the orderly timing of physiological functions across the day.

**Figure 1:**
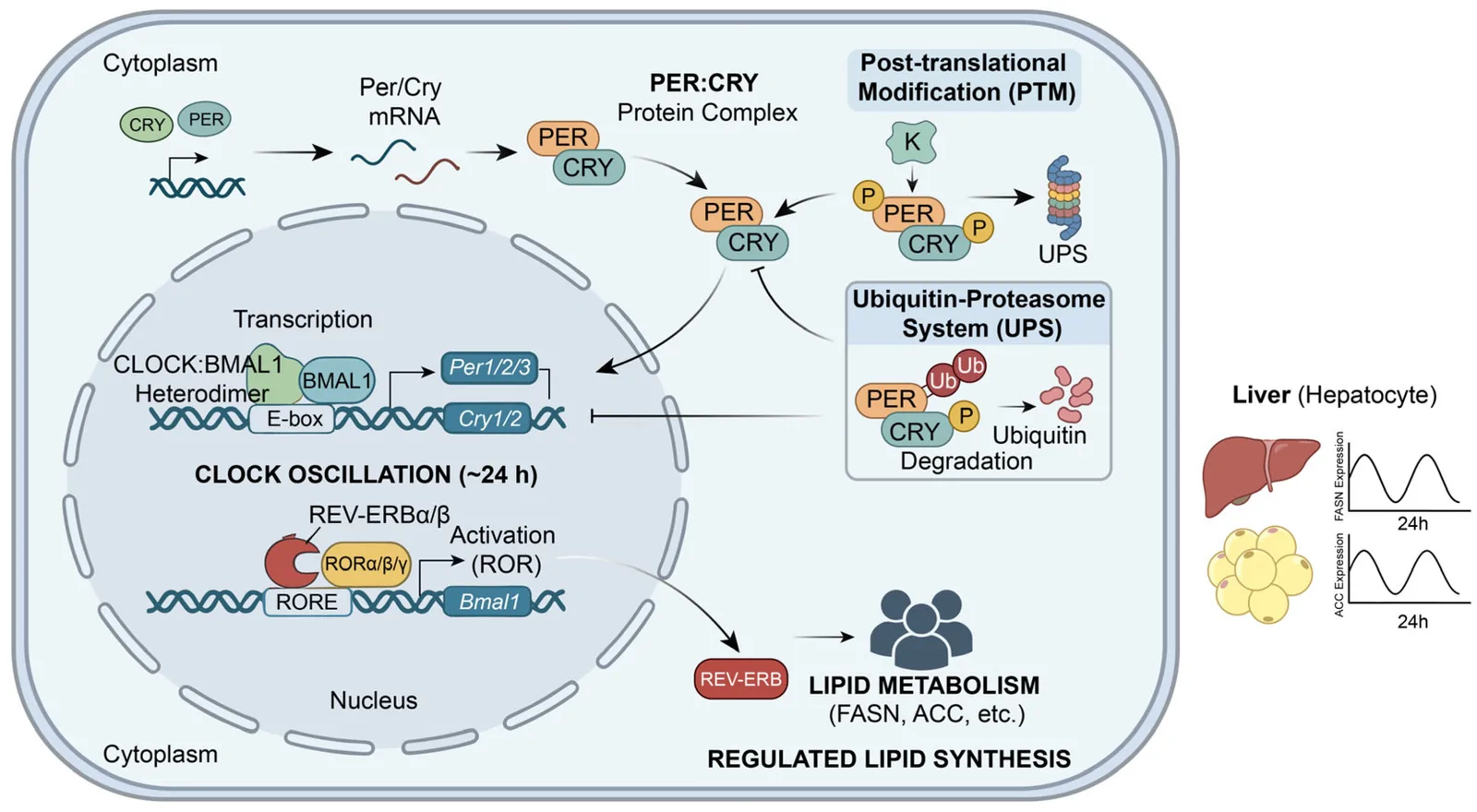
Molecular Mechanisms Regulating Mammalian Circadian Rhythms. The figure summarizes the core CLOCK–BMAL1/PER–CRY transcriptional–translational feedback loop, the auxiliary REV-ERB/ROR loop, and post-translational regulation of PER/CRY stability through phosphorylation and ubiquitin–proteasome-mediated degradation. These interconnected mechanisms generate approximately 24-h oscillations in clock gene activity and contribute to the rhythmic regulation of lipid metabolic pathways in peripheral tissues such as the liver and adipose tissue.

In mammals, the circadian system consists of the suprachiasmatic nucleus (SCN) in the hypothalamus and peripheral clocks, with rhythmic stability maintained through the periodic expression of clock genes and associated feedback mechanisms [11]. The circadian rhythms are primarily driven by transcriptional–translational feedback loops (TTFLs) [12]. The Circadian Locomotor Output Cycles Kaput and Brain and Muscle ARNT-Like Protein 1 (CLOCK–BMAL1) heterodimer binds to E-box elements within the promoters of target genes and promotes the transcription of core clock genes, including Period (Per) and Cryptochrome (Cry). Newly synthesized PER and CRY proteins gradually accumulate in the cytoplasm and subsequently translocate into the nucleus, where they repress CLOCK–BMAL1 transcriptional activity, thereby establishing a negative-feedback loop that generates near-24-h oscillations [13]. In parallel, an auxiliary feedback loop involving REV-ERB and ROR further regulates the expression of core clock genes. The nuclear receptor subfamily 1 group D members 1 and 2 (REV-ERBα/β) and RORα/β/γ compete for binding to ROR response elements (ROREs) in the promoters of target genes such as Bmal1, functioning as transcriptional repressors and activators, respectively. This mechanism contributes to the maintenance of rhythmic Bmal1 expression and enhances the stability of the core molecular oscillator [14,15]. Beyond transcriptional feedback, post-translational modification and degradation of clock proteins are also critical determinants of the period and amplitude of circadian oscillations. Phosphorylation of PER and CRY is regulated by kinases such as Casein Kinase 1 Epsilon/Delta (CK1ε/δ) and AMP-Activated Protein Kinase (AMPK), which in turn influence their stability, subcellular localization, and degradation. These proteins are subsequently targeted for degradation by the ubiquitin–proteasome system, progressively relieving negative feedback and allowing the next cycle of CLOCK–BMAL1-dependent transcription to begin [16]. REV-ERBα and REV-ERBβ, as key components of the secondary feedback loop, are themselves rhythmically expressed downstream of CLOCK–BMAL1 activation and function as transcriptional repressors that directly suppress Bmal1 transcription, thereby contributing to the regulation of circadian amplitude and phase [14] (Figure 1). Circadian rhythms are closely involved in a wide range of behavioral, physiological, and biochemical processes in animals, including the sleep–wake cycle, locomotor activity, mood regulation, and immune and endocrine functions [17,18]. Maintaining circadian stability is therefore important for normal tissue and organ metabolism as well as overall physiological homeostasis.

## 3. The Influencing Factors of Circadian Rhythm

### 3.1. Illumination Time

Environmental light serves as the dominant timing signal that synchronizes endogenous circadian oscillations with the external day–night cycle. Light signals are detected by the retina and transmitted to the SCN through the retinohypothalamic tract, where they reset the phase of the central clock and synchronize it with the external light–dark cycle [19]. Consistent alternation of light and darkness is critical for preserving temporal organization, metabolic stability, and the homeostatic balance of the gut microbiota [20,21]. Regular alternation between light and darkness also contributes to the temporal organization of feeding, neuroendocrine activity, metabolism, and gut microbial rhythmicity, as reported in previous studies [22,23]. When this temporal structure is disturbed, changes in host behavior and physiology may be accompanied by alterations in microbial composition and daily oscillation, providing a potential link between abnormal light exposure and disruption of the intestinal microbial ecosystem.

Altered light environments can disrupt the temporal and functional organization of the gut microbiota. Simulated jet lag and constant darkness have been linked to reduced microbial rhythmicity, whereas constant light can alter community structure and microbial functional activity [24,25]. For example, constant light increases the relative abundance of *Ruminococcus torques* while reducing *Lactobacillus johnsonii*, together with impaired intestinal barrier function [26]. Metatranscriptomic analysis in the same model identified altered expression of several microbial functional genes, including *hbd* (3-hydroxybutyryl-CoA dehydrogenase), *adhE* (acetaldehyde dehydrogenase), LptF (lipopolysaccharide export permease protein), and *kdsB* (3-deoxy-manno-octulosonate cytidylyltransferase), reflecting changes in microbial metabolic activity and lipopolysaccharide synthesis or transport [26]. More direct experimental evidence has also shown that dim light at night reduces the proportion of gut microbes displaying daily oscillation, while genetic elimination of intrinsically photosensitive retinal ganglion cells (ipRGCs) markedly attenuates microbial rhythmicity [27]. This points to a role for retinal light input in organizing gut microbial rhythms, although it remains unclear whether the associated microbial changes directly contribute to the metabolic abnormalities observed under disrupted light conditions.

In addition to the timing of light exposure, light intensity and spectral composition also influence circadian entrainment. The intensity and spectral characteristics of natural daylight vary throughout the day, providing light cues that are better aligned with physiological requirements and helping to maintain normal circadian amplitude and sleep–wake patterns [28,29,30]. In contrast, artificial light at night, light pollution, and shift work extend light exposure into biologically inappropriate periods, thereby increasing the risk of circadian disruption [31]. Blue light strongly stimulates intrinsically photosensitive retinal ganglion cells (ipRGCs). Morning exposure can facilitate circadian phase adjustment, whereas exposure at night or before bedtime can suppress melatonin secretion and alter fat oxidation and energy metabolism [32,33]. Prolonged exposure to abnormal light conditions may also disrupt microbial rhythmicity across distinct intestinal niches, including the cecum, intestinal lumen, mucus layer, and epithelial surface [24,34]. Current evidence indicates a clear association between abnormal light exposure and disruption of gut microbial rhythmicity, although mechanistic evidence remains derived largely from animal studies. It is still unclear whether these microbial changes directly contribute to light-induced metabolic dysfunction or primarily reflect secondary responses to host circadian disruption.

### 3.2. Dietary Composition and Circadian Rhythms

Dietary composition represents an important nutritional determinant of circadian regulation. Metabolic cues generated by different nutrients can act on peripheral tissues, including the liver and adipose tissue, to modulate clock gene expression and associated metabolic pathways [35,36]. Thus, the metabolic impact of diet is determined not only by nutrient type and proportion but also by the way peripheral clocks respond to these nutrient-derived signals.

Among dietary factors, the effects of high-fat diets on circadian regulation have been studied most extensively. High-fat feeding can markedly reshape the circadian transcriptional profile of adipose tissue, altering the rhythmic expression of numerous genes and pathways involved in lipid metabolism. Returning to a low-fat diet can restore part of this disrupted rhythmicity [37]. High-fat feeding can also disturb the circadian expression of clock and metabolic genes in the liver and adipose tissue and is accompanied by abnormalities in glucose and lipid metabolism [38]. Western-style diets, typically characterized by high fat and sugar intake and low dietary fiber, may further alter the composition and rhythmicity of the gut microbiota and exacerbate host metabolic dysfunction [39,40]. Beyond dietary fat, protein intake can also influence peripheral clocks. Recent studies have shown that different levels of dietary protein intake can modify PER2 oscillations across multiple peripheral tissues and affect the expression of clock-controlled and endoplasmic reticulum stress-related genes, while exerting comparatively limited effects on the central clock [41]. These findings suggest that the circadian effects of dietary composition are not unique to high-fat diets and that different nutrients may influence peripheral temporal organization through distinct metabolic and nutrient-sensing pathways. The gut microbiota also represents an important link between dietary composition and host rhythmicity. High-fat, low-fiber diets can alter the diurnal oscillation of the gut microbiota and its metabolites, whereas increasing dietary fiber intake may partially restore these rhythms [42]. For example, oat fiber has been shown to improve the rhythmic expression of hepatic clock proteins in high-fat diet-fed mice, accompanied by changes in gut microbiota-derived short-chain fatty acids (SCFAs) [6]. The influence of diet on circadian rhythms varies with tissue type, nutrient composition, and the state of the gut microbiota. How these factors interact to shape circadian regulation is still not fully resolved.

### 3.3. Meal Timing and Feeding Patterns

In addition to dietary composition, meal timing is an important temporal cue influencing circadian organization and metabolic homeostasis. Regular feeding–fasting cycles help synchronize peripheral tissues, including the liver, adipose tissue, and gastrointestinal tract, with the overall circadian system, whereas persistently delayed or irregular eating may promote misalignment between central and peripheral rhythms and increase the risk of obesity, type 2 diabetes, and cardiometabolic dysfunction [5,43]. The timing of food intake itself is also important. In general, concentrating a larger proportion of daily energy intake within the biologically active phase is associated with more favorable glucose utilization and energy metabolism, whereas evening or nighttime eating is more often linked to impaired glucose tolerance, abnormal insulin responses, and increased lipid storage [44,45]. Changes in meal timing can also affect peripheral clocks and the gut microbial ecosystem. Studies have shown that feeding during the inactive phase alters the rhythmic expression of hepatic clock genes, including Per1, Cry1/2, and Rev-erbα, and is accompanied by changes in the composition and diurnal oscillation of the gut microbiota [46]. These results indicate that meal timing influences not only when nutrients enter the body but also the temporal organization of peripheral tissues and the gut microbiota.

Time-restricted eating has received increasing attention as a chrononutrition intervention. In animal studies, the approach is generally referred to as time-restricted feeding (TRF), whereas time-restricted eating (TRE) is more commonly used in human studies. Both approaches confine daily food intake to a defined time window without necessarily reducing total energy intake. Animal studies have shown that, even under comparable energy intake, TRF can reduce obesity, hepatic steatosis, and insulin resistance while preserving or restoring rhythmic clock gene expression in peripheral tissues [47]. These findings indicate that meal timing itself can influence metabolic outcomes independently of differences in total energy intake. In human studies, TRE has been associated with reductions in body weight and visceral adiposity, together with improvements in some glycemic and lipid parameters, although findings have not been fully consistent across trials. The timing and duration of the eating window, baseline metabolic status of participants, and differences in study design are likely to influence the magnitude of the response [48]. Restricting food intake to a certain number of hours per day is therefore not sufficient to characterize the metabolic impact of a TRE regimen.

Beyond the duration of the daily eating window, the timing of the first and last meals may also influence metabolic responses. Earlier eating is generally associated with better glucose tolerance and insulin sensitivity, whereas habitual breakfast delay or frequent nighttime eating may weaken normal metabolic rhythms [49,50]. Observational studies have associated later eating with higher HbA1c levels and a greater risk of metabolic syndrome, whereas short-term intervention studies have shown that nighttime eating worsens postprandial glucose and insulin responses in both healthy individuals and patients with type 2 diabetes [51,52,53]. The duration of the eating window and its circadian timing therefore represent distinct aspects of meal timing. Even when two regimens allow a similar number of eating hours, their metabolic effects may differ if one eating window is positioned earlier and the other later in the day.

## 4. Circadian Rhythm Regulates Lipid Metabolism

### 4.1. Melatonin

Melatonin, often referred to as the “hormone of darkness,” is an endogenous hormone rhythmically synthesized by the pineal gland and secreted in a circadian manner. Its secretion typically peaks during the nighttime and is tightly regulated by the environmental light–dark cycle. Accordingly, melatonin is regarded as an important endocrine signal linking the circadian system to metabolic homeostasis [54]. Beyond its role in sleep–wake regulation and seasonal rhythmicity, melatonin is also closely involved in energy metabolism, body weight regulation, and immune function [54,55,56]. In metabolic tissues such as the liver, adipose tissue, and skeletal muscle, melatonin can modulate the rhythmic expression of clock and metabolism-related genes, thereby influencing lipid synthesis, lipid mobilization, and fatty acid oxidation [57]. These effects place melatonin at an important interface between circadian timing and peripheral metabolic regulation. The biological actions of melatonin are mediated primarily by the membrane receptors melatonin receptor 1 (MT1) and melatonin receptor 2 (MT2), both of which belong to the GPCR family and are widely expressed in tissues including the liver, adipose tissue, pancreas, and central nervous system [58,59]. Upon binding of melatonin to MT1 or MT2, downstream signaling networks involving Gi/Gq proteins are activated, leading to modulation of multiple pathways, including adenylyl cyclase (AC), phospholipase C (PLC), and phosphoinositide 3-kinase/protein kinase B (PI3K/Akt) signaling. MT1 signaling primarily engages the phospholipase C–inositol 1,4,5-trisphosphate–Ca^2+^ (PLC–IP3–Ca^2+^) axis to activate protein kinase C (PKC) and extracellular signal-regulated kinases 1 and 2 (ERK1/2), while suppressing cyclic adenosine monophosphate–cAMP response element-binding protein (cAMP–CREB) signaling. MT2 signaling can additionally modulate the cyclic guanosine monophosphate–protein kinase G (cGMP–PKG) pathway, together with MT1, integrate ERK1/2, CREB, and Akt signaling [60]. Melatonin may also contribute to lipid homeostasis by reducing oxidative stress, suppressing inflammation, and improving mitochondrial function [61,62]. RORα is likewise involved in circadian regulation and lipid metabolism; however, current evidence is insufficient to establish it as a direct nuclear receptor for melatonin, and its involvement is more likely to reflect indirect regulatory mechanisms [63,64,65]. This makes an indirect regulatory relationship between melatonin and RORα more plausible than a direct receptor interaction.

Melatonin also interacts with the gut microbiota. Experimental studies have shown that exogenous melatonin improves intestinal barrier function and alleviates microbiota disturbances induced by sleep disruption, weaning stress, or high-fat feeding. In some settings, these effects are accompanied by improvements in lipid metabolism and insulin sensitivity [66,67,68,69]. In some animal models, melatonin-induced changes in the gut microbiota and intestinal barrier function are accompanied by improvements in lipid metabolism and insulin sensitivity. Gastrointestinal melatonin appears to act mainly in local regulation of intestinal barrier function, microbial ecology, and metabolism, whereas its contribution to circulating melatonin levels and whole-body circadian regulation remains controversial [69,70]. Factors such as artificial light at night, shift work, social jetlag, and late eating may also alter the rhythm of melatonin secretion and create misalignment between melatonin signaling and feeding–metabolic cycles [71]. Nocturnal light exposure has been associated with lower melatonin levels in a meta-analysis [72]. Persistent temporal misalignment may further disrupt peripheral metabolic rhythms and is associated with increased risks of obesity, dyslipidemia, and other metabolic disorders [73]. The involvement of the gut microbiota appears to depend on the experimental context, and its contribution to melatonin-related metabolic regulation is currently supported more strongly by animal intervention studies than by human evidence.

### 4.2. Leptin

Leptin is a key anorexigenic hormone secreted primarily by white adipocytes, and its circulating concentration is closely associated with adipose tissue mass. As an important humoral signal, leptin links peripheral energy stores to central nervous system control [53]. Leptin acts mainly through the hypothalamic leptin receptor (LepR), activating the Janus kinase 2–signal transducer and activator of transcription 3 (JAK2–STAT3) signaling pathway to suppress appetite and regulate energy expenditure, thereby contributing to lipid metabolism and energy homeostasis [74]. Leptin secretion also exhibits a pronounced circadian pattern, with circulating levels generally rising at night and declining in the early morning. This rhythm is influenced by the sleep–wake cycle, meal timing, and the local circadian clock in adipose tissue [75,76]. Leptin therefore conveys not only information about energy stores but also a temporally organized metabolic signal.

Circadian disruption can alter the phase and amplitude of leptin secretion and interfere with normal leptin signaling. Shift work, sleep deprivation, and forced desynchrony have been associated with altered circulating leptin levels and reduced leptin sensitivity [76]. In db/db mice with severely impaired leptin signaling, hyperphagia and obesity are accompanied by widespread disruption of tissue rhythmicity, supporting a role for intact leptin signaling in the temporal coordination of energy metabolism [77]. More recent evidence indicates that the succinate receptor 1 (SUCNR1) in adipocytes can modulate leptin expression through circadian mechanisms and influence energy metabolism under different nutritional states, further linking the adipose tissue clock to leptin regulation [78,79]. The gut microbiota may also influence leptin signaling. In Western diet-fed animals, depletion of the gut microbiota has been shown to reduce hypothalamic inflammation and improve leptin responsiveness, partly through GLP-1 receptor signaling [80]. Microbial metabolites, including short-chain fatty acids, may also affect leptin expression and secretion in adipocytes. However, these effects vary with adiposity, diet, and host metabolic status, making it difficult to define a single underlying mechanism [81]. The microbial contribution to leptin regulation is therefore likely to depend on metabolic context rather than operate through a single conserved pathway. Circadian disruption and leptin resistance may reinforce one another at the level of energy regulation, while the extent to which the gut microbiota participates in this interaction remains less well defined.

### 4.3. Gut Microbiota

**Figure 2:**
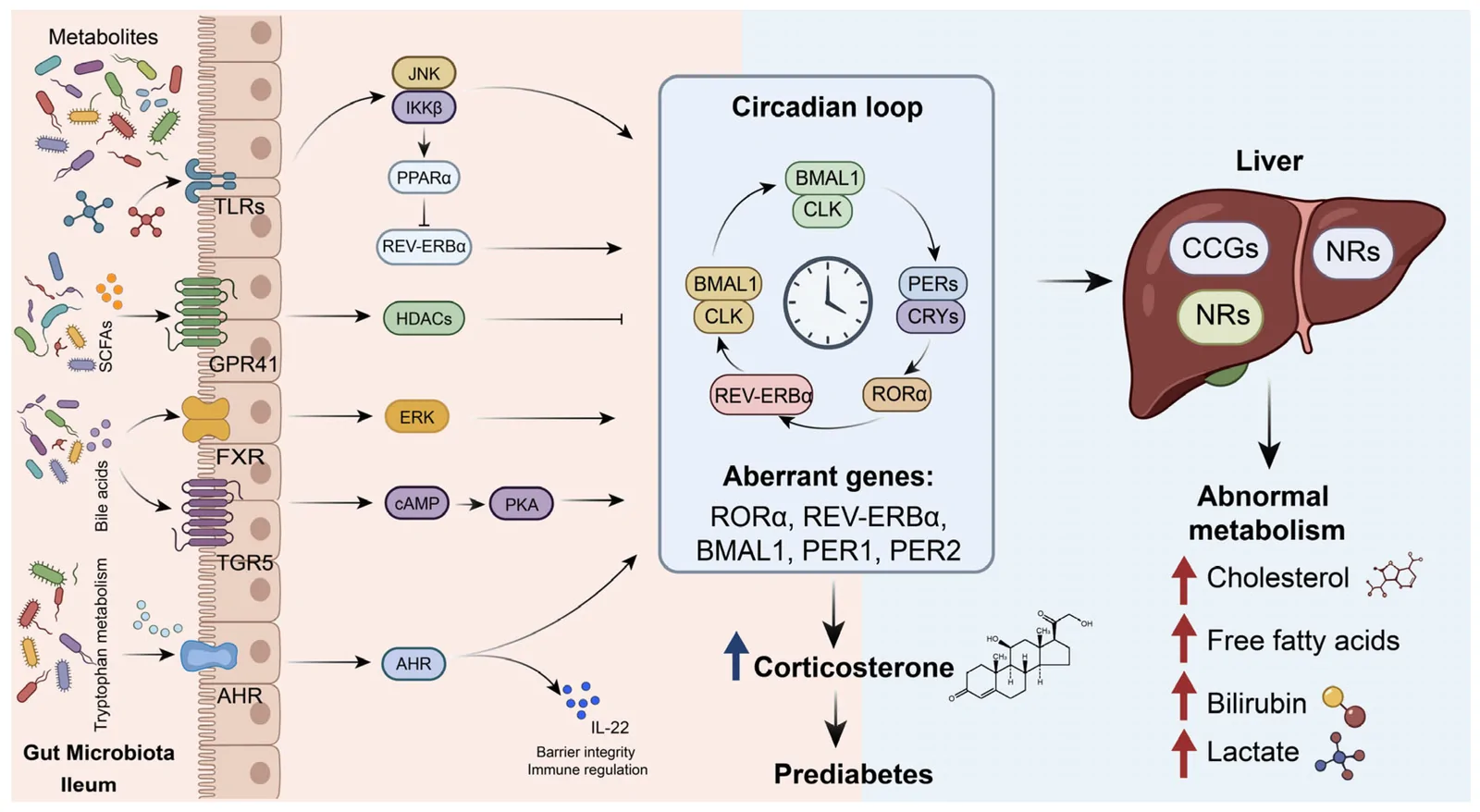
Gut Microbiota–Clock Crosstalk in Circadian and Metabolic Homeostasis. The figure summarizes how microbial signals and metabolites influence the host circadian clock through several pathways. TLR signaling modulates the JNK/IKKβ–PPARα–REV-ERBα axis, while SCFAs, bile acids, and tryptophan metabolites act through GPR41/HDAC-, FXR/TGR5-, and AhR-related pathways, respectively. These signals converge on core clock components, including BMAL1/CLOCK, PER/CRY, REV-ERBα, and RORα, and can subsequently alter hepatic clock-controlled genes, nuclear receptor signaling, corticosterone levels, and metabolic profiles.

The gut microbiota is an integral component of host metabolic regulation and plays a critical role in the maintenance of circadian rhythms and lipid metabolism [82]. Under the combined influence of circadian timing, nutrient availability, and host physiological state, the intestinal environment and substrate availability change over the course of the day, giving rise to diurnal variation in microbial composition, function, and metabolite production [83,84]. Changes in microbial community structure and metabolic activity may in turn feed back on host metabolic rhythms. Across multiple studies, circadian disruption has been associated with gut dysbiosis, impaired lipid and glucose metabolism, and an increased risk of metabolic disorders such as obesity and type 2 diabetes [82,85,86]. This bidirectional relationship places the gut microbiota within the broader network that coordinates host circadian and metabolic regulation (Figure 2).

Germ-free animal studies provide relatively direct experimental evidence supporting a role for the gut microbiota in host circadian regulation. Compared with conventionally raised mice, germ-free mice show altered expression of clock-related genes in the liver and central circadian system under both low- and high-fat dietary conditions [87]. The microbiota itself also displays clear temporal organization. Time-series studies in mice have identified multiple bacterial taxa with approximately 24-h oscillations, each characterized by distinct amplitudes and peak phases; high-fat feeding dampens many of these rhythms, whereas restricting food intake to the active phase can partially restore them [88,89]. The host circadian clock also contributes to the maintenance of microbial rhythmicity. Microbial oscillations persist under constant darkness, whereas intestinal epithelial-specific deletion of Bmal1 markedly disrupts these rhythms [90]. Several bacterial taxa show reduced or lost 24-h rhythmicity in individuals with type 2 diabetes, accompanied by changes in related microbial metabolic pathways [91]. The temporal organization of the gut microbiota involves not only changes in abundance, but also variation in oscillation amplitude and peak phase, both of which are shaped by diet, the host circadian clock, and metabolic state. Host–microbiota interactions can interface with circadian networks through multiple signaling pathways. Toll-like receptors (TLRs) expressed by intestinal epithelial cells sense microbial signals and participate in the rhythmic activation of JNK and IKKβ; this process can further influence PPARα-mediated regulation of REV-ERBα, thereby altering local circadian rhythms and the expression of metabolism-related genes [92]. Gut microbiota depletion has also been shown to alter the expression of clock-related genes, including RORα, REV-ERBα, BMAL1, PER1, and PER2, together with increased ileal corticosterone levels and disturbances in glucose metabolism [87]. Similar alterations have been observed in the livers of germ-free mice, including changes in clock-controlled genes and several metabolism-related nuclear receptors, accompanied by altered levels of cholesterol, free fatty acids, bilirubin, and lactate [93].

In addition to host receptor signaling, microbiota-derived metabolites provide a major route through which the gut microbiota communicates with peripheral circadian systems. Short-chain fatty acids (SCFAs), particularly acetate, propionate, and butyrate, exhibit clear diurnal variation and are linked to rhythmic changes in peripheral clock function [7]. Experimental studies provide more direct evidence for this relationship. In microbiota-depleted mice, oral administration of SCFAs shifted the phase of PER2 rhythms in the liver, kidney, and submandibular gland in a time-of-day-dependent manner [94]. In the colon, daily fluctuations in fecal SCFA levels parallel rhythmic expression of the SCFA receptors FFAR2 and FFAR3, while disruption of Bmal1 abolishes SCFA rhythmicity and attenuates several downstream physiological effects [95]. Microbiota-derived SCFAs can therefore contribute to the regulation of peripheral circadian clocks. However, comparable phase-shifting effects were not observed in isolated liver slices or fibroblasts, suggesting that this response may depend, at least in part, on systemic or inter-tissue signals present in the intact organism rather than being entirely cell autonomous. Bile acids represent an important signaling link among gut microbial metabolism, circadian regulation, and lipid metabolism. Primary bile acids synthesized in the liver enter the intestine, where they can be deconjugated by microbial bile salt hydrolases and further transformed by other microbial enzymes into a range of secondary bile acids, thereby altering the composition and signaling properties of the bile acid pool [96]. Different bile acids can regulate lipid, glucose, and energy metabolism through receptors such as FXR and TGR5 [97]. At the same time, microbial modification of bile acids can influence host clock-related gene expression. Experimental studies have shown that unconjugated bile acids can alter the expression of core clock and clock-controlled genes in intestinal epithelial cells as well as in the intestine and liver of mice [98]. Feeding-time interventions further showed that changes in the diurnal bile acid profile occur together with coordinated shifts in the rhythms of hepatic clock genes, bile acid synthesis-related genes, and lipid metabolism genes [46]. These findings support a role for bile acid metabolism in transmitting signals among the gut microbiota, peripheral clocks, and lipid metabolism, although the specific contributions of individual bile acids and their receptors remain incompletely defined. Tryptophan metabolism represents another temporally organized route of microbiota–host communication. Gut bacteria can convert dietary tryptophan into indole and a range of indole derivatives, several of which act on host signaling pathways involved in intestinal barrier function, immune regulation, and metabolic control [99]. One well-characterized example is indole-3-aldehyde produced by Lactobacillus species, which activates the aryl hydrocarbon receptor (AhR) and promotes IL-22 production, thereby enhancing mucosal defense [100]. Tryptophan metabolism itself also exhibits pronounced diurnal variation. Studies using germ-free and antibiotic-depleted mice have shown that disruption of the gut microbiota alters the daily pattern of intestinal tryptophan metabolism, while shotgun metagenomic analysis revealed that most bacterial species displaying rhythmicity also possess tryptophan-metabolizing capacity [101]. Time-resolved studies have further identified circadian oscillations in several circulating tryptophan metabolites, together with temporal variation in AhR activity [102]. The diurnal variation in tryptophan metabolism further indicates that microbial metabolic output is itself temporally organized and may contribute to the dynamic regulation of host circadian signaling.

The relationship between the gut microbiota and host circadian rhythms is reflected not only in changes in microbial composition, but also in the daily oscillation of microbial communities and their metabolic activity. Studies using germ-free animals, microbiota depletion, and metabolite interventions have shown that some microbial signals can directly influence peripheral clocks and metabolic processes, whereas human studies have mainly identified associations between altered microbial rhythmicity and metabolic phenotypes. It therefore remains difficult to determine whether these rhythmic changes contribute to metabolic dysfunction or arise mainly as a consequence of disrupted host circadian regulation.

### 4.4. Energy Management

Energy metabolism and circadian rhythms are reciprocally regulated. The circadian clock coordinates nutrient uptake, storage, and utilization so that lipid synthesis and oxidation occur in an orderly manner across different times of day. In turn, changes in cellular energy status can feed back on the molecular clock [103]. Consistent with this bidirectional regulation, many genes involved in lipid metabolism in the liver, adipose tissue, and skeletal muscle exhibit pronounced circadian oscillations, and disruption of these rhythms is frequently accompanied by abnormalities in fatty acid synthesis, β-oxidation, and triglyceride metabolism [5,104,105]. Loss or dysfunction of clock genes can increase susceptibility to obesity and disturbances in glucose and lipid metabolism, whereas prolonged metabolic imbalance can further weaken normal circadian organization [106]. This reciprocal relationship depends on cellular systems that continuously sense nutrient availability and energy status.

Among these systems, sirtuin 1 (SIRT1), AMP-activated protein kinase (AMPK), and mechanistic target of rapamycin (mTOR) provide major points of communication between metabolism and the molecular clock. SIRT1 activity is closely linked to nicotinamide adenine dinucleotide (NAD^+^) availability, which itself undergoes circadian oscillation [107,108,109]. During fasting or energy restriction, increased SIRT1 activity suppresses lipogenesis, promotes fatty acid oxidation, and supports mitochondrial function [110,111,112]. AMPK responds primarily to cellular energy deficiency: an increased AMP/ATP ratio activates AMPK, which can modify circadian timing by regulating the stability of CRY1 and PER2. AMPK-mediated phosphorylation promotes CRY1 ubiquitination and degradation and can also influence PER2 stability through CK1ε-related mechanisms [113]. By contrast, mTOR responds predominantly to nutrient abundance and interacts with clock components including CLOCK, BMAL1, and CRY1 while coordinating protein synthesis, lipogenesis, and cell growth [104,114]. Through these complementary sensing pathways, fasting and nutrient abundance are translated into distinct signals that can modify clock function and metabolic output.

Peroxisome proliferator-activated receptor γ coactivator 1α (PGC-1α) provides an additional link between circadian regulation and mitochondrial metabolism. PGC-1α can enhance RORα-dependent transcription and participate in the regulation of clock genes such as BMAL1, while also promoting mitochondrial biogenesis, oxidative metabolism, and fatty acid utilization [115,116]. SIRT1, AMPK, mTOR, and PGC-1α therefore connect cellular energy status with the temporal control of lipid metabolism through distinct but interacting mechanisms. Energy metabolism is consequently not simply an output of the circadian clock: nutrient availability and cellular energy state can feed back on clock function, while circadian disruption can disturb the normal timing of lipid synthesis, oxidation, and storage. Persistent loss of this temporal coordination favors lipid accumulation and related metabolic abnormalities.

## 5. Circadian Rhythm and Obesity

Obesity is not solely associated with chronic excess energy intake and insufficient physical activity; circadian disruption is also increasingly recognized as an important contributing factor [104]. Epidemiological and clinical studies have linked long-term shift or night work, insufficient sleep, social jetlag, and irregular daily schedules with weight gain, central obesity, type 2 diabetes (T2DM), and increased cardiometabolic risk [117,118,119,120]. Abnormal light exposure and impaired clock gene function can promote weight gain and disrupt glucose and lipid metabolism [114,121,122]. The metabolic consequences of circadian disruption therefore extend beyond body weight regulation and involve a broader loss of temporal coordination across metabolic tissues.

Under physiological conditions, lipogenesis, lipolysis, glucose utilization, and energy expenditure follow distinct temporal patterns that align nutrient storage and use with the activity cycle [123,124]. When central and peripheral rhythms become misaligned, this temporal organization is disrupted, and the balance between nutrient utilization and storage may shift accordingly. Reduced metabolic flexibility and increased lipid storage may therefore emerge as persistent circadian misalignment develops. Adipose tissue is a major metabolic target of circadian disruption. The adipocyte clock contributes to the temporal regulation of lipogenesis, lipolysis, and adipokine secretion. Disruption of these rhythms is commonly accompanied by adipocyte hypertrophy, altered fatty acid mobilization, low-grade inflammation, and reduced insulin sensitivity [122]. These disturbances can increase the burden of circulating free fatty acids and exacerbate metabolic stress in other tissues. Hepatic metabolism also exhibits distinct temporal organization; processes such as fatty acid β-oxidation, cholesterol synthesis, and bile acid metabolism are regulated by circadian rhythms and involve transcriptional regulators such as REV-ERBα, PPARα, and SREBP-1c [115,125]. A loss of this temporal organization can disrupt the balance between lipid synthesis and oxidation, thereby promoting hepatic lipid accumulation and the development of fatty liver phenotypes [126]. Reduced hepatic metabolic flexibility may also aggravate systemic disturbances in glucose and lipid metabolism [127]. Consequently, hepatic metabolic dysfunction can extend the metabolic consequences of circadian disruption beyond the liver.

**Figure 3:**
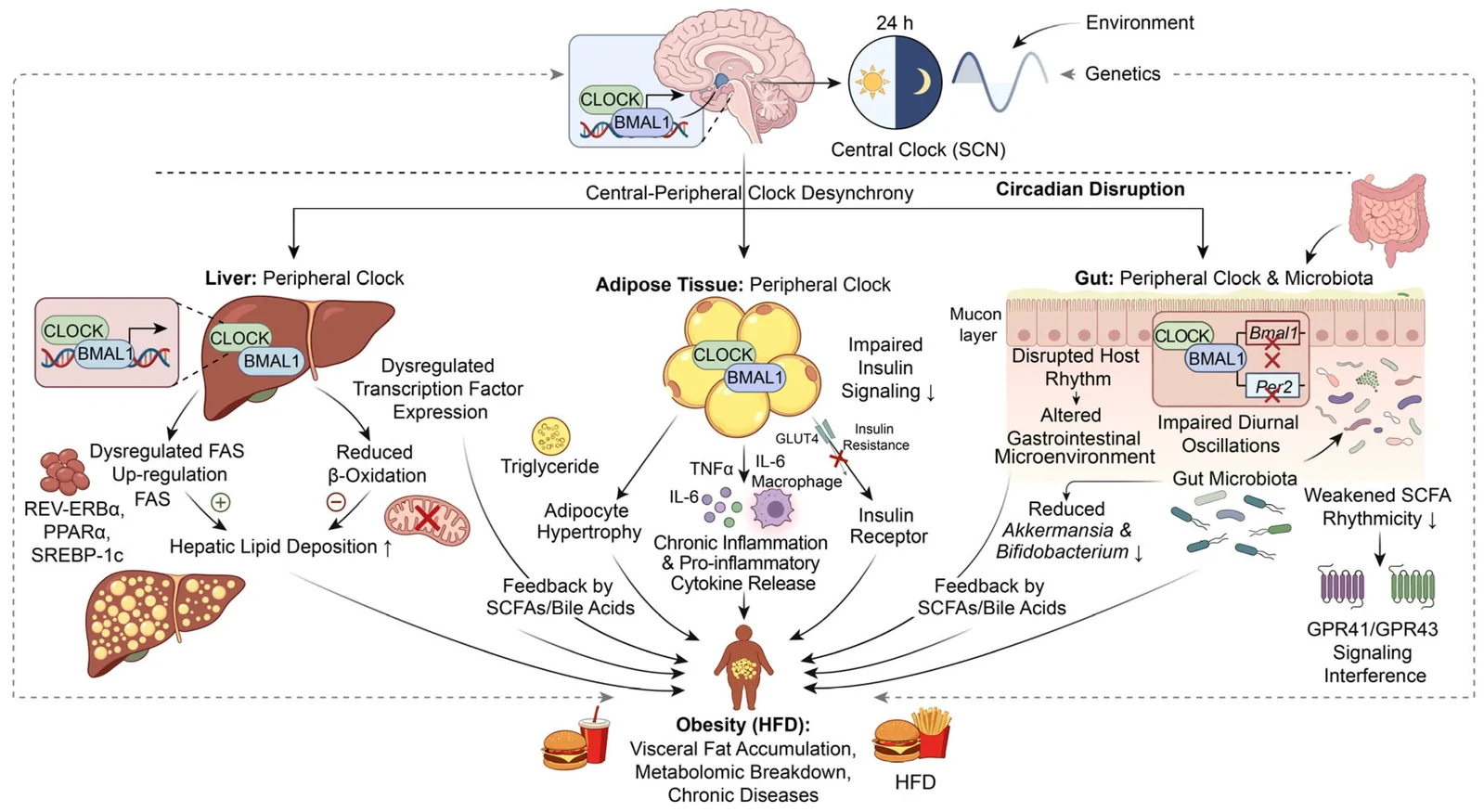
Circadian Disruption, Obesity, and Metabolic Dysfunction. This figure illustrates the metabolic changes associated with desynchronization between the central and peripheral clocks in the liver, adipose tissue, and gut. In the liver, these changes include increased lipogenesis, reduced β-oxidation, and lipid accumulation, whereas adipose tissue is characterized by adipocyte hypertrophy, impaired insulin signaling, and chronic low-grade inflammation. At the same time, alterations in host and microbial rhythms, together with changes in the intestinal microenvironment and microbial metabolites, can further disturb metabolic homeostasis. These combined changes favor visceral fat accumulation and obesity-related metabolic dysfunction.

The gut microbiota adds another layer to the relationship between circadian disruption and obesity. In obesity and high-fat diet models, microbial rhythmicity is often dampened or reorganized, together with changes in community composition, intestinal barrier function, and microbial metabolic activity [89,122,128]. These alterations are associated with low-grade inflammation, insulin resistance, and disturbed lipid metabolism, but human studies do not yet establish whether they contribute directly to obesity or largely arise as a consequence of the altered metabolic state. Intervention studies in animals provide stronger support for causal involvement in some settings, although these effects remain dependent on diet, host metabolic phenotype, and environmental context. The gut microbiota is therefore better viewed as a potential modulator linking host rhythmicity with metabolic phenotype rather than as a single causal driver of obesity (Figure 3).

The metabolic consequences of circadian disruption ultimately reflect a progressive loss of temporal coordination across multiple tissues and systems. Persistent misalignment among adipose tissue, the liver, the gut microbiota, and neuroendocrine signals can disturb the balance among lipid storage, oxidation, and utilization. Although current evidence consistently supports an association between circadian disruption and obesity risk, its independent contribution in humans remains difficult to separate from diet, sleep, and other lifestyle factors, an issue that will require further longitudinal and intervention studies.

## 6. Conclusions

Over the past few decades, our understanding of mammalian circadian rhythms and their relationship with metabolic homeostasis has continued to expand. In this review, we use the gut microbiota as an entry point to examine how light exposure, feeding time, dietary composition, hormonal signals, and energy metabolism influence circadian organization and lipid metabolism, with particular attention to the role of the gut microbiota in linking host rhythmicity with lipid homeostasis. Current evidence suggests that the circadian clock, host metabolism, and gut microbiota are connected through continuous bidirectional communication that helps maintain metabolic balance. When this coordination is chronically disturbed, lipid homeostasis may be impaired, thereby increasing the risk of obesity, type 2 diabetes, cardiovascular disease, and other metabolic disorders. Despite substantial progress, the key links among circadian regulation, microbial metabolic activity, and host lipid metabolism remain incompletely understood. Future studies should further clarify the causal relationships among these factors and evaluate the practical value of strategies such as time-restricted feeding, light management, and microbiota-targeted interventions in the prevention and treatment of metabolic diseases.

## Data Availability

No new data were created or analyzed in this study. Data sharing is not applicable to this article.

## Abbreviations

The following abbreviations are used in this manuscript:

SCN	Suprachiasmatic Nucleus
BMI	Body Mass Index
SREBP-1C	Sterol Regulatory Element-Binding Protein 1c
SCFAs	Short-Chain Fatty Acids
TTFLs	Transcriptional–Translational Feedback Loops
CLOCK	Circadian Locomotor Output Cycles Kaput
BMAL1	Brain and Muscle ARNT-Like Protein 1
PER	Period Circadian Regulator
CRY	Cryptochrome Circadian Regulator
ROR	Retinoic Acid Receptor-Related Orphan Receptor
REV-ERBα	Nuclear Receptor Subfamily 1 Group D Member 1
REV-ERBβ	Nuclear Receptor Subfamily 1 Group D Member 2
ROREs	ROR response elements (ROREs)
CK1ε/δ	Casein Kinase 1 Epsilon/Delta
AMPK	AMP-Activated Protein Kinase
TAG	Triacylglycerol
FASN	Fatty Acid Synthase
ACC	Acetyl-CoA Carboxylase
SCD	Stearoyl-CoA Desaturase
PGC-1β	Peroxisome Proliferator-Activated Receptor γ Coactivator 1β
GPCR	G protein-coupled receptor
TRF	Time-restricted feeding
TRE	Time-Restricted Eating
PPAR	Peroxisome Proliferator-Activated Receptor
ACADs	acyl-CoA dehydrogenases
SUCNR1	Succinate receptor 1
MT1/2	Melatonin Receptor 1/2
AC	Adenylyl Cyclase
PLC	Phospholipase C
PKC	Protein Kinase C
ERK1/2	Extracellular Signal-Regulated Kinase 1/2
ROS	Reactive Oxygen Species
NAFLD	Non-Alcoholic Fatty Liver Disease
HFD	High-Fat Diet
SIRT1	Sirtuin 1
NAD^+^	Nicotinamide Adenine Dinucleotide
mTOR	mechanistic target of rapamycin
PGC-1α	Peroxisome proliferator-activated receptor γ coactivator 1α
T2DM	Type 2 diabetes
